# Abstract of the Proceedings of the Buffalo Medical Association

**Published:** 1864-09

**Authors:** Joseph A. Peters

**Affiliations:** Secretary


ART. II—*Abstract of the Proceedings of the Buffalo Medical Association.*

TUESDAY EVENING, August 2d, 1864.

Present—Drs. White, Rochester, Lockwood, Miner, Congar, Gay, Strong, Ring, Johnson, Smith and Peters. In the absence of the President Dr. Strong was called to the Chair. There were no minutes to read.

*Dr. Lockwood* said the Association having discussed for the past few months uterine diseases, tumors, and similar subjects, he thought that the introduction of something fresh might produce an agreeable variety, hence he begged leave to present a specimen of the *Apocynum Cannabinum*, or Indian Hemp, which he had procured for the purpose of calling the attention of the Association to what he considered a valuable article of the materia medica. Had often been joked about his fondness for the use of "roots and herbs," and would confess that he estimated very highly the importance of studying our indigenous remedies, and was very glad to make use of them on proper occasions. Wished to call particular attention to the characteristics of this species, as it was often confounded with the Dog-bane [*A. Androsœmifolium*] which was much more violent, and even poisonous in its action. The Indian Hemp, as would be seen, had an erect, herbaceous stem, branching at top, and in the present instance about four feet high; the leaves are opposite, oblong-ovate, acute at both ends, and downy underneath; the flowers are small, of a reddish or pinkish tinge, and grow in cymes; the fruit consists of long pointed pods or receptacles, containing numerous imbricated seeds, each furnished with a long seed-down. The plant grows in moist situations, in the skirts of woods or fences, and flowers from June to August. When wounded it emits a milky juice, and it has a tough, fibrous bark, from which it takes its name of Indian Hemp. Would call particular attention to the color of the flowers, which furnished the surest and almost the only means of distinguishing it from Dog-bane, which had white flowers. The root is the portion used in medicine, and may be given either in decoction or extract. The decoction is made by boiling half an ounce of the powdered root in a pint and a half of water until a pint remain, of which a table spoonful is a dose for an adult. The medium dose of the extract is one grain. Thought it usually given in too large doses. Had been in the habit of prescribing this remedy for thirty years, and valued it very highly for its diuretic and alterative action, in connection with its properties as a hydragogue cathartic. From one to two grains of the extract he had found in many cases to stimulate the liver as manifestly as the mercurials. But had found it chiefly valuable in anasarca following scarlet fever, as well as in the dropsy which often attacked old men debilitated from dissipation. Indeed he had come to place great reliance on it in all cases of dropsy, but would advise it to be used with caution where there was any tendency to heart disease, as it possessed in a great degree the property of lessening the force of the heart's action. Neglect of this caution might cause mischief. In conclusion he hoped that what he had said might induce physicians who had an opportunity to observe its action in hospitals to give it a trial.

*Dr. Rochester* expressed his thanks to Dr. Lockwood for the pains he had taken to present this specimen to the Association, and especially for the valuable distinction pointed out by him between that and the Dog-bane. Was glad to be able to confirm to a marked degree all the Doctor had said with reference to its value in dropsy. It is mentioned by both Wood and Stille, as a valuable remedy, and he had himself found it so in a great many cases. Recollected particularly a case of a boy who had ascites following remittent fever, and who had taken iron and other remedies without effect, but upon whom the *apocynum* had a happy effect. Was taught a lesson once by having given it to a patient with organic disease of the heart. The patient did not die, but he would say most emphatically that *it depresses*, and should never be given in such cases.

*Dr. White* had been much interested by the remarks of Dr. Lockwood, and would confess that the remedy was nearly new to him, as he had never used it. Had however had the opportunity of seeing its efficacy in a case of which Dr. Lockwood had the care, and which, as the Doctor had been too modest to mention it, he would briefly refer to. The patient was a man with cirrhosis of the liver, and ascites to an extent truly enormous. He had been seen by, and in the care of several physicians of the town, but without much apparent benefit, but coming under Dr. L.'s treatment recovered. Was inclined to think *apocynum* valuable in such cases.

*Dr. Miner* was greatly interested by the subject presented by Dr. Lockwood, and had no doubt he had presented a valuable remedy. Did not however suppose any specific effect for it in dropsy could be claimed, that it was a valuable cathartic, but no more of a specific than others. Elaterium had a similar action, as he would illustrate by a case in point. A young lady was admitted to the Hospital of the Sisters of Charity, where she remained a short time, and left it for a private boarding house, where he saw her. Her menstruation had stopped, there was obstinate vomiting, great prostration, pulse was from 130 to 160; respiration was impeded; the abdomen was distended, and near the stomach a hard, nodulated tumor could be detected, having very much the feeling of scirrhus. As there was undoubtedly fluid in the abdomen paracentisis was made, and from eight to ten quarts of water drawn off. The patient had taken nearly all cathartics, but without having caused a movement of the bowels for days; he now administered to her a half grain of elaterium, which produced copious vomiting, and the evacuation *per anum* of a pail full of fœcal matter. The tumor disappeared after this copious evacuation, showing it to have been an accumulation of hardened fœces. Considered the case interesting, both on account of the effect produced by the elaterium, and because of the location of the accumulation in the upper part of the colon, which was in his experience rare—it usually occurring lower down. Had no doubt that any active cathartic would have proved as efficient; perhaps the remedy presented by Dr. Lockwood might have answered a similar purpose.

*Dr. Congar* remarked that he prescribed for the case while in the Hospital of the Sisters of Charity one-sixth gr. of elaterium every four hours. The patient only remained a few hours, and was taken away by her friends; presumed it was the same case treated by Dr. Miner.

*Dr. Rochester* thought Dr. Miner a little sceptical in regard to medicines; thought there were some specific effects in remedies, and that we should look for these effects if we would improve our therapeutics. Thought fœcal accumulations much more frequent in the colon than in the rectum, as the rectum was much more easily evacuated than the colon. Would refer to an article on this subject by Dr. Batchelder in the *New York Journal of Medicine.* These accumulations were often mistaken for tumors. Injections of biborate of soda had been recommended for them.

*Dr. Miner* would say that *such* accumulation as he had described in the colon, was very unusual. Hardened fœcal matter might lodge for a time in the colon, but it was rarely in quantity to be discovered. The exceptional cases where it has been detected have been regarded as worthy of record and have thus found place in the journals. The rectum, though easily evacuated, is still so frequently found filled with impacted fæces, that mention is not made of it as in any way unusual or remarkable ; numerous cases fall under the observation of every practitioner, while in his opinion not one in a hundred ever meet with it in the colon, in quantity sufficient to attract attention.

*Dr. Lockwood,* said he did claim for the Indian Hemp more than a cathartic effect. Considered it to have what he must call an alteratic effect though a certain distinguished physician had said that term ought never to be used. Referred to the case mentioned by Dr. White to say that the man had entirely recovered.

*Dr. Ring* related a case of obstinate constipation in a lady 39 years of age. At the time he saw her first she had had no movement of the bowels for nineteen days, although fifteen drops, in all, of croton oil had been given her. Found a tumor on the region of the colon which exhibited considerable tenderness on pressure. Fearing Peritonitis he prescribed calomel and opium, with the application of a sinapism to the abdomen. After a few days the symptoms of inflammation having subsided, attempts were made to procure a passage *per anum* and he finally succeeded after repeated injections of soap and water, using an injection pipe twenty-two inches in length in order to reach as near the obstruction as possible. On the thirtieth day she had a full evacuation of scybala, and convalesced pleasantly.

Reports on Prevailing diseases being called for :

*Dr. Gay* had seen a good deal of cholera infautum and diarrhœa.

*Dr. Rochester* had seen a good many cases of what was more than merely cholera morbus. He referred to a dysentery or serous diarrhœa which might be called cholerine and which would undoubtedly be called Asiatic cholera were that disease prevailing. Had in mind one lady who was taken at 3 A. M. and when he called at 9 he found her apparently moribund. Thought there was a morbid agent in the atmosphere which would be likely to result, in the autumn, in the production of dysentery. Had seen, as he thought, an illustration of this in two cases which he related. The first was that of a carpenter who was taken in the afternoon with diarrhœa, having four evacuations, about eleven o'clock he was found dead on the bed. Post mortem revealed the fact that he had organic disease of the heart, but not in sufficient degree, probably to have caused his death at that time alone. He probably died from the combined effect of the heart disease and the lowering of vitality caused by this morbific agent. The same night a man in the same neighborhood died under similar circumstances, the only lesion found on *post mortem,* was concentric hypertrophy of the heart.

*Dr. White* could hardly agree with Dr. Rochester in the belief that there was any special morbid agent in the atmosphere. Had noticed only the usual diseases of the season, a little aggravated perhaps by the extreme dryness and heat of the season. The thermometric average for July was 3° higher than had been known here before, since records were kept.

*Dr. Miner* had seen considerable diarrhœa and cholera morbus, some cases so severe that in a time of the prevalence of cholera it could not be distinguished from that disease. Dr. Rochester's cases of heart disease were interesting, especially as concentric hypertrophy had been almost excluded from the list of diseases upon the discovery that the contraction of the ventricle occurring *in articulo mortis* might give to the heart the appearance of concentric hypertrophy. That it does sometimes occur there can be no doubt. Its great rarity and the fact that its existence as a possible state is doubted by some careful observers, makes Dr. Rochester's cases of great interest, and suggests the possibility of error since the examination was not made by Dr. R. with a view to this point. As to any epidemic influence, uniting to cause death, he would only remark, that sudden deaths were often unaccountable in our present knowledge.

*Dr. Smith* related a very interesting case which had recently occurred in his practice, and which certainly very strongly simulated cholera.

*Dr. Strong* thought the tendency to serous diarrhœa which certainly existed, could be accounted for by the influence of the extremely hot and dry weather, especially on the contents of our sewers, which, instead of being washed out by rains had been accumulating their contents and baking in the sun.

Similar reports were made by Drs. Lockwood, Gay, Congar, Ring and Johnson.

The Society, after transacting some miscellaneous business adjourned.

JOSEPH A. PETERS, Secretary.

